# Bioactive Compounds, Pharmacological Actions, and Pharmacokinetics of Genus *Acacia*

**DOI:** 10.3390/molecules27217340

**Published:** 2022-10-28

**Authors:** Gaber El-Saber Batiha, Nosheen Akhtar, Abdulrahman A. Alsayegh, Wafaa Fouzi Abusudah, Najlaa Hamed Almohmadi, Hazem M. Shaheen, Thakur Gurjeet Singh, Michel De Waard

**Affiliations:** 1 Department of Pharmacology and Therapeutics, Faculty of Veterinary Medicine, Damanhour University, Damanhour 22511, Egypt; 2 Department of Biological Sciences, National University of Medical Sciences, Islamabad 46000, Pakistan; 3 Clinical Nutrition Department, Applied Medical Sciences College, Jazan University, Jazan 82817, Saudi Arabia; 4 Clinical Nutrition Department, College of Applied Medical Sciences, Umm Al-Qura University, Makkah 24381, Saudi Arabia; 5 Chitkara College of Pharmacy, Chitkara University, Punjab 140401, India; 6 Smartox Biotechnology, 6 rue des Platanes, 38120 Saint-Egrève, France; 7 L’institut du Thorax, INSERM, CNRS, Université de Nantes, 44007 Nantes, France; 8 LabEx «Ion Channels, Science & Therapeutics», Université de Nice Sophia-Antipolis, 06560 Valbonne, France

**Keywords:** *Acacia*, phytochemicals, pharmacological properties, antidiabetic, antioxidant

## Abstract

Plants are a promising source of bioactive compounds that can be used to tackle many emerging diseases both infectious and non-infectious. Among different plants, *Acacia* is a very large genus and exhibits a diverse array of bioactive agents with remarkable pharmacological properties against different diseases. *Acacia*, a herb found all over the world, contains approximately more than 1200 species of the Fabaceae family. In the present review, we have collected detailed information on biochemical as well as pharmacological properties. The data were retrieved using different databases, such as Elsevier, PubMed, Science Direct, Google Scholar, and Scopus, and an extensive literature survey was carried out. Studies have shown that *Acacia* possesses several secondary metabolites, including amines, cyanogenic glycosides, flavonoids, alkaloids, seed oils, cyclitols, fluoroacetate, gums, non-protein amino acids, diterpenes, fatty acids, terpenes, hydrolyzable tannins, and condensed tannins. These compounds exhibit a wide range of pharmaceutical applications such as anti-inflammatory, antioxidant, antidiarrheal, antidiabetic, anticancer, antiviral, liver protective effects, and so on. Thus, the literature shows the tremendous phytochemical impact of the genus *Acacia* in medicine. Overall, we recommend that more research should be conducted on the medicinal value and isolation and purification of the effective therapeutic agents from *Acacia* species for the treatment of various ailments.

## 1. Introduction

With more than 1200 species, the genus *Acacia* is the second largest in the Leguminosae (Fabaceae) family. The genus is distributed throughout warm tropical and temperate regions of the world, with the greatest number of species (about 957) in Australia and a significant number (around 185) in America, Africa (144 species), and Asia (89 species). [1]. Many pharmacological molecules have been isolated from diverse species of *Acacia*. The root, leaves, pods, and bark of *Acacia* hold the uppermost quantity of tannin and phenolic compounds, such as gallic acid, dicatechin, quercetin, robidandiol, β-amyrin, hentriacontane, betulin, sitosterol, kaempferol-3 chlorogenic acid, and glucoside isoquercetin [2]. *A. nilotica* (L.) Willd. Ex Del., which is recognized as a medicinal tree, belongs to the family Mimosaceae and has been identified as being abundant in phenolic substances involving gallic acid, condensed tannin, and also phlobatannin, epigallocatechin-7-gallate, and (+)-catechin, and has been used for the treatment of amoeboid dysentery, viral-induced colds, bronchitis, bacterial-induced diarrhea, bleeding piles leukoderma, and fungal diseases [3]. The flowers and leaves of this perennial tree are also used as animal fodder. It has been shown previously in the Genetic Toxicology Laboratory that *A. nilotica* plants had cytotoxic and antimutagenic activities [4]. *Acacia* extracts were documented for their cytotoxicity effects, antioxidant activities [5], and antimalarial effects [6]. In another study, isorhamnetin 3-o-neohesperidoside was identified from the leaves *of A. laeta* and was reported to protect the cells against oxidative stress through the inhibition of the enzyme xanthine oxidase. Moreover, it is documented to scavenge a superoxide anion with an inhibition capability against *Staphylococcus aureus*, *Klebsiella pneumonia*, *Klebsiella oxytoca*, and strains [7]. Similarly, the number of other secondary bioactive metabolites identified from several *Acacia* species has been reported for their pharmacological potential [8]. Our aim is to provide a comprehensive review of the chemical components and pharmaceutical aspects of medicinally active *Acacia* species. Table 1 summarizes the significant activities of different species of *Acacia*. We have used different databases, including Science Direct (http://www.sciencedirect.com, accessed on 30 May 2022), PubMed (http://www.ncbi.nlm.nih.gov/pubmed, accessed on 25 May 2022)), and Google Scholar (http://scholar.google.com, accessed on 30 May 2022)).

## 2. Chemical Components Isolated from *Acacia*

*Acacia* is a varied genus including a variety of bioactive components such as alkaloids [33], flavonoids [8], terpenoids [34], phenolic compounds, and tannins [35,36], which are accountable for various pharmacological and biological properties such as antibacterial, anti-inflammatory, antihypertensive, antiplatelet, hypoglycaemic, anti-atherosclerotic, analgesic, and anticancer, owing to their higher free radical scavenging and antioxidant properties [37]. The phytochemical analysis of the *A. nilotica* leaf extracts revealed that it contains volatile oil, saponins, hydrolysable tannin, flavonoids, tannins, triterpenoid, phenol, alkaloids which are actual important compounds when regarding the pharmacologically bioactive phytochemicals in the plant [38]. Members inside the genus *A. sensu lato* were documented to enclose cyclitols, cyanogenic glycosides, amines, essential oils, diterpenes, simple alkaloids, fatty acids of the fluoroacetate, seed oils, triterpenes, amino acids, saponins, phytosterols, gums, flavonoids, and hydrolyzable reduced tannins as well. Deboshree Biswas and M.G Roymon reported that the bioactive principles found in methanolic bark and leaf extracts of *A. arabica* were investigated using an HPLC/MS/MS. Oleic acid (C_18_H_34_O_2_), Ferulic acid (C_10_H_10_O_4_), Myristic acid (C_14_H_28_O_2_), Palmitic acid (C_16_H_32_O_2_), Quercetine 3-O- (4′-O-acetyl)-rhamnopyranoside (C_28_H_30_O_16_), p-Coumaroyl-glucoside (C_15_H_19_O_7_), p-Coumaroylquinic acid (C_16_H_18_O_8_), and Steroidal sapogenin were the most common chemicals found in leaf and bark extracts. Caffeic acid phenethyl ester (CAPE) (C_17_H_16_O_4_), Methyl 3,4,5 tri hydroxyl benzoate, and Epi catecine-3-gallate (C_22_H_18_O_10_), were also found in the leaf extracts (C_8_H_8_O_5_). 3,4,5-trihydroxybenzoate was one component isolated from a bark extract (C_7_H_6_O_5_). Overall, this genus (and other mimosoid legumes) looks to be deficient in acetylenes, coumarins, phenylpropanoids, anthraquinones, naphthoquinones, lignans, glucosinolates, stilbenes, and uncommon fatty acids. However, only a small number of species have been specifically examined for these substances; thus, undoubtedly, the phytochemicals of only a small portion of *Acacia* species have been examined yet. Various phytocompounds from different *Acacia* species are shown in Table 1.

### 2.1. Alkaloids and Amines

In most of the taxa comprising the genus *A. sensu lato*, both relatively simple alkaloids and amines are present. They are particularly plentiful in the seeds from the subgenus *Aculeiferumsection monacanthea* of the *Neotropical species*, and also the species of Africa, including *A. kraussiana*, *A. schweinfurthii*, *A. caesia*, *A. brevispica*, and *A. pentagona*. Moreover, many of the non-protein amino acids that are present in other plants are missing and habitually have N-methyltyramine [39]. Amines are also found in the vegetative parts of these plants in a majority [39]. Once sheep eat the fodder of the species of subgenus guajillo, *Aculeiferumis A. berlandieri*, from North America over prolonged periods, N-methyl-β-phenethylamine (PEA) produces hind-limb ataxia [40]; this compound may also decrease the fertility of male Angora goats [33]. N-Methyl-β-PEA (**1**) co-exist with β-PEA, N-methyltyramine in the vegetative material of guajillo [40], and three additional amines (hordenine, tyramine, and N-methyltyramine) [33]. Nonetheless, additional sensitive investigation using GC/MS showed the existence of minor quantities of almost 33 other alkaloids and amines, including mescaline, methylamphetamine, amphetamine, mimosine methyl esters, isoquinoline alkaloids, nicotine, and nornicotine [33]. *A. roemeriana* and *A. greggii* have both been shown to contain N-Methyl—PEA; however, it is not limited to that subgenus. This component also occurs in *A. angustissima* of series Filicinae, *A. rigidula*, *A. constricta*, and *A. schottii*. Tyramine is established in *A. angustissima*, *A. roemeriana*, and *A. greggii* [40]. Β-PEA and the associated amines have been correspondingly described in certain Australian species of subgenus Phyllodineae involving *A. cultriformis*, *A. hakeoides*, *A. harpophylla*, *A. floribunda*, *A. kettlewelliae*, *A. adunca*, *A. linifolia*, *A. lunata*, *A. podalyriaefolia*, *A. longifolia*, *A. pravissima*, *A. suaveolens*, and *A. prominens* [41]. N-methyltryptamine likewise N, N-Dimethyltryptamine, and other N-methylated tryptamines are present in *A. maidenii*, *A. simplicifolia*, and *A. phlebophylla* bark [42]. Inside the root bark of *A. spirorbis* and leaves of *A. harpophylla*, hordenine exists, while N-α-cinnamoylhistamine is present in *A. polystacha*, *A. argentea*, leaves, and *A. spirorbis* fresh fruit bark [41].

### 2.2. Cyanogenic Glycosides

Several *Acacia* species possess substances of cyanogenic glycoside nature and are able to deliver hydrogen cyanide once the plant tissue is attacked. For this to happen, the β-glycosidases and the glycosides must be together. Of the identified cyanogenic species, plants have been defined as either devoid of both the enzyme and the substrate, possessing both of them, or having either one of them. Animals may be poisoned by the latter variety. Cyanogens have been discovered in *Acacia* plants in over 70 species [43], including 45 of the subgenus Phyllodineae and 25 of the subgenus *Acacia*. A small number of taxa in the subgenus Aculeiferum have been shown to contain cyanide. Cyanogens of the *Acacia* subgenus are a group of aliphatic components originated from leucine, isoleucine, valine, and, in particular, linamarin, proacipetalin, lotaustralin, epiproacipetaline, proacaciberin, heterodendrin, and 3-hydroxyheterodendrin [44]. Australian species (Almost 96%) in the subgenus Phyllodineae have been explored in a survey. The results showed that 45 species were found to be positive for cyanogenicity, and most of them lie in the Juliflorae group [45]. In these, the cyanogenic glycosides include prunasin (**6**) and sambunigrin (**7**). However, two species, i.e., *A. pulchella var. reflexa* and *A. exilis*, of the Phyllodineae subgenus have been shown to contain heterodendrin, an aliphatic cyanogen [46]. Generally speaking, cyanogenic species in the Phyllodineae subgenus lack the enzymes required to hydrolyze the compounds rapidly. Though the cyanogenic species of *Acacia* are widely distributed in Australia, few reports of poisoning livestock attributed to *Acacia* species exist [45]. The African species *A. hereroensis* and *A. caffra* of the subgenus *Aculeiferum* also encompasses the aromatic cyanogens, sambunigrin, cyanogens, and prunasin. In addition, herbarium specimens of *A. welwitschia*, *A. klugii*, and *A. chariessa* were weakly positive for the release of hydrogen cyanide following the Feigl–Anger test.

### 2.3. Hydrocarbons

To date, 13 hydrocarbons (**111**–**123**), mostly isolated from *A. modesta* and *A. nilotica* species, have been reported in the genus *Acacia*. The presence of various compounds in the chloroform extract of *A. nilotica* leaves was revealed by mass spectroscopy and gas chromatography analysis, such as palmitic acid, linolenic acid, lariciresinol, myristic acid, stearic acid, 2-methylresorcinol acetate, 1,3,4 eugenol, megastigmatrienone, neophytadiene, δ-5-avenasterol,3,4,7-trimethylquercetin, and arachidonic acid [47]. In addition, a benzene extract of the stem bark of *A. modesta* contains betulin, α-amyrin, γ–sitosterol, and octacosanol. The plant’s heartwood extracts in pet. ether (60–80°) and alcoholic extracts yielded γ–sitosterol and pinitol, respectively. The extract of leaves in pet. ether (60–80°) yielded hentriacontane, octacosane, hentriacontanol, and octacosanol [48].

### 2.4. Fatty Acids

The seed oils derived from the separates of *A. sensu lato* vary slightly in their biochemical content. Linoleic (LA) and oleic (OL) acids prevail in the oil of seeds of many species, even though a few comprise moderately high proportions of linolenic acids. Most oils have stearic and palmitic acids in the residual portion, and most species possess 3–10% oil inside their seeds. The fatty acids of the seed oil from species of subgenus comprise: *A. farnesiana* (linolenic acid; 54%, LA; 43% and OL; 13%); *A. caven* (linoleic acid (LA): 54%); A. giraffae (oil; 3.5%, with stearic acid, palmitic acid, palmitoleic, and n (41.5%)) [8]; *A. macrothyrsa* (21% OL acid; 50% LA acid (LA)); *A. lenticularis* (80% linolenic acid); *A. modesta* (27% OL; 26% LA); A. leucophloea (32% OL; 38% LA); *A. nilotica* (29% OL acid; 44.5% LA); *A. seyal* (36% OL acid; 38% LA); *A. planifrons* (34% OL; 23% LA); *A. sieberiana* (31% OL; 44% LA); *A. arabica* (42% OL; 36% LA), and *A. tortilis* (19% OL, 72% linolenic acid, and 60% LA); [49]. The *A. lenticularis, A. farnesiana, A. tortilis*, and *A. nilotica* seeds possessed coronaric acid (cis-9,10-epoxyoctadec-cis-12-enoic) (9) [50]. *A. giraffae* contains stearic, palmitic, palmitoleic, OL, and arichidic acid (29%) in their seed pod oils [51]. The acids from the seed oils of subgenus Aculeiferum include: *A. mellifera* (22% LA; 42% OL acid); *A. catechu* (29% OL; 41% LA) [52]; *A. polyacantha* (26% OL acid, 41% LA); *A. senegal* (10% OL acid, 43% LA, and 36% stearic acid); *A. suma* (77% linolenic acid); and *A. sinuata* (30% OL; 36.8% LA), *A. schweinfurthii* (23% OL acid, 36% LA). Coronaric acid (9) is present in *A. pennata* seeds, while vernolic acid (cis-12,13-epoxyoctadec-cis-9-enoic) (10) is found in the seeds of *A. mellifera*, *A. catechu*, and *A. sinuata* [49]. The seed oil of *A. willardiana* has 18% OL acid and 37% LA [53]. Aboriginal people used the seeds of Australian wattle species (subgenus Phyllodineae) as sources of oils and protein [54]. Those vital for these species for ‘bush tucker’ in Australia are *A. murrayana* and *A. victoriae* (20% protein, 18% saturated fatty acids, 18% OL, 5% oil, and 62% LA) [49]. From the 20 other species of this subgenus, Aboriginal people use the oil content of18, enclosed from triglycerides (3 to 22%) in which either OL or LA preponderates [49]. The composition of oil from other members of subgenus Phyllodineae is *A. dealbata* (10% oil with 31% OL and 40% LA), *A. auriculiformis* (10% OL and 67% LA), *A. mearnsii* (8% oil with 17% OL and 68% LA), and *A. decurrens* (8%) [55]. The seed oil from *A. cyclops* (dry material (10%) of the seeds) was extremely unsaturated, while the funicle oil (40.6%) involved ordinarily saturated fatty acids [56]. Coronaric acid (**9**) is a part of *A. cochlearis* seeds, while *A. auriculiformis* contains vernolic acid (**10**) [55]. The *Faidherbia albida* seed oils remained comparable to those of common *Acacia* species. They comprised 23% OL acids and 51% LA, and coronaric acid (**9**) is also present in the seeds of this species [55].

### 2.5. Gums

Gum arabic, also known as gum *Acacia*, is seen as the main exudate extracted, with desired properties, generally from a particular African species, such as *A. senegal* (Aculeiferum). This gum is produced artificially in Sudan by injuring trees and gathering gum tears [56]. Approximately 250 g/tree/year is the average yield, and the whole annual production is close to 60,000 tons [57]. This component is hydro-soluble up to 50% by weight, resulting in a transparent mucilaginous solution with a low viscosity. Gums are produced in response to injury, stress, bacteria, insects, or fungal attacks on the plant as dispersible complex carbohydrates. [58]. Gums are usually released from damaged sites into fissures and fractures in the bark or appear as uneven masses or “tears” on the surface of branches or trunks. Even though the configurations of naturally occurring vegetal gums differ widely, several containing gum arabic have backbones of d-galactopyranose units linked 1 → 3, and many contain four sugars: l-rhamnose, d-glucuronic acid and l-arabinose, and d-galactose. They comprise the cations Mg^2+^, Ca^2+^, and K^+^ [56]. Gums exhibiting subgenus *Acacia* comprise *A. arabica*, *A. nebrownii*, *A. adansonii*, *A. calcigera*, *A. giraffae*, *A. drepanolobium*, *A. nubica*, *A. chundra*, *A. farnesiana*, *A. ehrenbergiana*, *A. gerrardii*, *A. hebeclada*, *A. heteracantha*, *A. hockii*, *A. kirkii*, *A. leucophloea*, *A. nilotica*, *A. karoo*, *A. rigidula*, *A. reficiens*, *A. sieberiana*, *A. seyal*, *A. xanthophloea*, and *A. tortilis* [59]. Subgenus *Aculeiferum* comprise: *A. catechu*, *A. cheiranthifolia*, *A. goetziisubsp.goetzii*, *A. berlandieri*, *A. campylacantha*, *A. erubescens*, *A. fleckii*, *A. leucospira*, *A. laeta*, *A. Senegal*, and *A. mellifera* [60]. Several species of *Acacia* subgenus Phyllodineae are able to produce large amounts of gums [61]. Amongst the species formerly used as commercial gums, Australian black wattle (*A. dealbata*, *A. decurrens*, *A. pycnantha*, *A. homalophylla*, or *A. sentis*,) belong, and the gum of an unnamed wattle species too [62]. In the early 1900s, *A. rivalis* gum provided the foundation for a commercial gum manufacturing industry in South Australia [63]. Nevertheless, a large number of these Australian commercial gums are known to have poor quality; strong in taste, were dark reddish brown vis à vis the color was more likely to alter in gels than in mucilage with water. Gums grown east of the Great Dividing Range are mostly not soluble in water; however, the ones with dry inner are likely to be more water-soluble [64]. In other new investigations, the chemical and physical characteristics of many wattle gums have been studied [59]. Though the initial investigations showed that gums from the species of the Phyllodineae subgenus were described with a low rhamnose percentage, low acidity, high galactose/arabinose ratio, and low intrinsic viscosity, advanced studies have shown much more dissimilarity in several parameters than those firstly observed [59]. The characteristics of common gums from the *Acacia* species are shown in Table 2.

### 2.6. Non-Protein Amino Acids

Non-proteinous amino acids are usually found in leaves and seeds throughout the species of the subfamily Mimosoideae [74]. Nearly 100 *Acacia* species have been investigated, and the majority of them had non-protein amino acids. [39]. These components can be hazardous to non-adapted animals as well as humans who consume them. N-acetyldjenkolic acid (**11**) is consistently present in members of subgenus *Acacia*. It was first isolated in *A farnesiana* [75]. Utmost enclosed α-amino-β-oxalylaminopropionic acid (**12**) (a neurolathyrogen) and free α,β-diaminopropionic acid [39]. N-4-oxalyl-2,4-diaminobutyric acid (**13**), 2,4-diaminobutyric acid, and 2-amino-6-N-oxalylureidopropionic acid (oxalylalbizziine) (**14**) are also present in the seeds of these species [76]. Several possess 2,4-cis-4,5-trans-dihydroxypipecolic acid (**15**). Nonetheless, most of the amino acids stated above are missing Neotropical species seeds of the subgenus Aculeiferum Monacanthea, and in African species *A. caesia, A. brevispica*, *A. kraussiana*, *A. schweinfurthii*, and *A. pentagona*. However, they have amines such as N-methyltyramine [39]. Despite the fact that *A. coulteri* seeds include all of the characteristics of the subgenus *Aculeiferum sensu stricto*, this species belongs to a group that has been proven to be distinct from other members of this subgenus using molecular techniques [77]. *A. coulteri* do not have N-acetyldjenkolic acid in their seeds. The seeds of *A. millefolia* and *A. willardiana*, species related to *A. coulteri*, enclose S-[β-carboxyisopropyl]-l-cysteine and willardiine (**16**) (**17**) [78]. Willardiine is rarely present in additional mimosoid legumes [78]. The seeds of *A. angustissima* (may be *A boliviana*, series Filicinae) cover the non-protein amino acids 2,4-diaminobutyric acid, 2-amino-4-acetylaminobutyric acid, and 2-amino-6N-oxalylureidopropionic acid (oxalylalbizziine) (**14**) [76]. Inside the seeds of *A.lemmonii* (a segregate of *A. angustissima*) willardiine is also found (**16**) [78]. This series’ amino acid pattern is distinct from that of the other *A. sensu lato* groups [76]. The combination of S-carboxyethylcysteine, S-[-carboxyisopropyl]-l-cysteine (**17**), albizziine (**18**), and amino-acetylaminopropionic acid (**19**) are found in over 60 species of the Phyllodineae subgenus.) [39]. Pipecolic acid (**20**), as well as hydroxypipecolic acid (**21**), are present in the leaves *of A. mearnsii* (as considerably as 1% dry weight) [79]. Hydroxypipecolic acid (**21**) is also present in the heartwood of *A. maidenii* [80]. Furthermore, the non-protein amino acid content of *F. albida* seeds, traditionally thought to belong to the *Acacia* genus, is comparable to that of subgenus *Aculeiferum* sensu stricto. The essential oils obtained from the *A. farnesiana flowers* (sweet *Acacia* or cassie ancienne) have been extensively used in the production of perfumes [81]. This species is cultivated in several tropical and subtropical countries for this reason and represents a significant harvest in southern France [82]. The volatile compounds were formerly isolated through enfleurage, which is the solvent extraction commonly applied [82]. Three compounds, trans-3-methyldec-4-enoic acid, cis-3-methyldec-3- en-1-oic acid, and cis-3-methyldec-3-en-1-ol, play a striking role in the aroma of cassie oil [81]. Cassie blossom, absolute of *A. farnesiana* flowers, encloses geranial (2.8%), methyl salicylate (47.5%), geraniol (9.8%), anisaldehyde (17.3%), geranyl acetate (3.3%), α-ionone (0.4%), 3-methyldec-3-en-1-ol (1.9%), benzaldehyde (6%), 3-methyldec-4-en-1-ol (0.5%), (β-ionone (0.7%), Z)-3-nonen-1-ol (0.7%), myrcene (0.5%), linalool (0.4%), and a quantity of other compounds [83]. *A. caven* flowers are also utilized in fragrances. However, the fragrance is appraised to be less desirable compared to *A. farnesiana* [84]. The composition of essential oils derived from the flowers of *A. rigidula* (subgenus *Acacia*) as well as *A. berlandieri* (subgenus Aculeiferum) was studied too. The major components from the essential oil identified in *A. rigidula* were: jasmone, cis3-hexenyl benzoate, p-anisaldehyde, citronellyl acetate, methyl 2,6-dihydroxybenzoate, and kaur-16-ene. Those identified in *A. berlandieri* were: 1-octanol, benzyl benzoate, linalool oxide B, and eugenol [83]. The essential oils of *A. floribunda* (subgenus Phyllodineae) and *A. dealbata* were removed using petroleum ether to yield concrete [84].

### 2.7. Terpenoids

Two types of cassane diterpenes (**22**;**23**) were identified in the *A. jaquemontii* roots [85]. The exudates and resinous leaves of *A. rossei* (subgenus Phyllodineae) in Western Australia have been shown to contain labdane diterpenes (**24**;**25**) [86]. The pods of *A. sinuata* (syn. A. concinna) (subgenus Aculeiferum) have been found to contain acacic acid lactone, machaerinic acid, and sapogenin B. Acacidiol, a nortriterpene, was also documented from the same source [87]. This was a novel 21-hydroxy ester molecule derived from monoterpenes. Furthermore, saponins were identified from this specie as well [87]. A genin, a triterpene ester, acacigenin B (**29**), was previously isolated from the same source [87]. The phytosterols -spinasterol (**30**) and stigmast-7-enol have been identified in a variety of Phyllodineae species, including *A. auriculiformis*, *A. meansii*, *A. maidenii*, *A. melanoxylon*, *A. sparsiflora*, and *A. obtusifolia* [88]. A phytosterol, α-spinasterol (**30**), and a triterpenoid trisaccharide, Acaciaside (31), have been described from *A. auriculiformis* [88]. Three triterpene glycosides were extracted from the leaves of A. myrtifolia: myrtifoliosides A, B, and C [89]. Many saponic (triterpene) glycosides derived from *A. victoriae* have recently been studied for their ability to induce apoptosis and reduce tumor cell growth [90]. The compounds termed Avicins (for example, Compound **34**, avicin D) inhibit tumor cell growth by disrupting mitochondrial activity [90]. In both entire carcinogenesis and initiation/promotion model systems, these drugs reduced papilloma formation in mice by 70% [91].

### 2.8. Flavonoids

Numerous flavone glycosides and flavonol, flavan-3, 4-diols, flavan-3-ols andaglycones, are present in the heartwood, leaves, and bark of *Acacia* species. In these flavonoids, the 5-hydroxy group is usually absent, which identifies the Fabaceae family. Commonly, the barks have considerable complex flavonoid mixes compared to the heartwoods [92]. The flavonoid content of the heartwoods and barks of the 61 native Australian *Acacia* species were examined by paper chromatography and broadly divided into four groups based on the variations in the phenolic hydroxyl pattern (3′,4′,7-trihydroxy; 4′,7-dihydroxy; 3′,4′,7,8-tetrahydroxy, or 4′,7,8-trihydroxy) [92]. The red-brown heartwood of *A. giraffae* holds (+)-mollisacacidin [(+)-7, 3, 4-trihydroxy-2, 3-trans-3, 4-trans-diol or earlier (+)-leucofisetinidin], which is connected to proanthocyanidins (PACs) [93]. Additionally, from this specie, an array of isomeric mollisacacidin byproducts have been described [94]. The heartwoods of *A. nilotica* subsp. kraussiana and *A. karroo*, both African species of this subgenus, contain a 7,3,4-substitution pattern. *A. luederitzii* vars. luederitzii and *A. reficiens* subsp. reficiens, and retinens have both 7,4-substitution and 7,3,4 pattern [95]. The flavonoids were extracted from the heartwoods of the subgenus Aculeiferum, e.g., *A. nigrescens*, rostrata, and *A. senegal* vars. Leiorachis exhibit a 7,8,34-substitution, while those of *A. galpinii, A. burkei*, and *A. erubescens* have a a 7,8,4-substitution pattern, and those in *A. welwitschii* have a pattern of 7,3,4 [95]. The Indian species of wood, *A. catechu*, encloses flavonoids with 5,7,3,4-hydroxylation, epicatechin, and catechin [80]. A 7,3,4-trihydroxyflavan-3,4-diol and many additional components with the above-mentioned pattern of substitution have been identified in the *A. angustissima* (*Acacia* series Filicinae) leaves [96]. The members of the Phyllodineae subgenus have a wide range of flavonoid types in their bark and leaves. The flavan-3-ols (+)-catechin (**45**) and (+)-gallocatechin (**47**) are common in the woods and barks of 61 Australian species of the subgenus Phyllodineae, but (-)-epicatechin (**46**) and (-)-epigallocatechin (**47**) are not common and often only occur in little quantities. The (+)-epicatechin and (+)-epigallocatechin gallate esters are uncommon, but they are abundant in some species. [92]. Flavonoids containing 7-hydroxy A-ring substituents are found in the Brunioideae, Uninerves sect. Racemosae, and Botryocephalae families of flavonoids. 3-Methoxyflavones are common in Australian heartwood. [80]. *8-O-methylflavan-3*, *4-diols* are found in *A. saxatilis* and *A. cultriformis*, while *3-O-methylflavan-3*, *4-diols* are found in *A. saxatilis* and *A. mearnsii* [80]. These components have been recognized since they are involved in a number of other members of Australian species [95].

### 2.9. Tannins

Some *Acacia* species, including those in the subgenera of Aculeiferum and *Acacia*, as well as those in the series Filicinae and other mimosoid legumes, contain hydrolyzable tannins [44]. Using the potassium iodate method for gallotannins, the leaves of various species and, to a lesser extent, their bark were found to contain between 1 and 8% hydrolyzable tannins [35]. The chemical 1,3-di-O-galloyl-4,6-hexahydroxydiphenoyl-glucopyranose has been found in the leaves of *A. raddiana* [97].

Condensed tannins, also referred to as proanthocyanidins (PACs), are the most noticeable secondary metabolites in the majority of *Acacia* species. Tannins are a vital part of various foods and provide great benefits in animal diets. The number of condensed tannins in the bark and leaves of American *Acacia* subgroups (subgenera *Acacia* and *Aculeiferum sensu stricto*, the series of Filicinae, and the species associated with *A. coulteri*) typically range from 1 to 8% (rare samples have reached 20%); typically, the wood samples have 1% or fewer tannins [35]. Mimosa (*Acacia mearnsii*) bark extracts are the major industrial sources of PACs [98,99]. PACs with 7,3,4- and 5,7,3,4-phenolic substitution configurations are found in the African species red-brown dark heartwood, i.e., *A. giraffae* of the *Acacia* subgenus. The above-mentioned PACs habitually have a (4β → 8)-linkage with a (+)-catechin unit. *A. luederitzii* also contains this type of tannin, but the species is dominated by (+)-guibourtinidol-(46)-(+) fisetinidol (**55**), (+)-guibourtinidol-(**46**)-, and (+)-(**46**)-epifisetinidols [100]. This source’s compounds usually have a 5-oxygenated ‘lower’ flavan-3-ol unit, such as (+)-catechin (**45**), (+)-epicatechin (**46**), or (+)-afzelechin (**47**). The bark of many Kenyan *Acacia* species has a moderately higher content of tannin: *A. mearnsii* (28.8%) (Subgenus Phyllodineae), *A. Senegal* (25.1%) (Subgenus Aculeiferum), *A. hockii* (24.1%), *A. mellifera* 19.3%, *A. kirkii* (16.1%), *A. nilotica* ssp. indica (11.6%), *A. xanthophloea* (17.0%) (subgenus *Acacia*), *A. nilotica* ssp. subulata (13.1%), *A. seyal* var. fistula (13.3%), 9.3% in *A. polyacantha* ssp. Campylacantha, and *A. sieberiana*, 4.7% [101]. In the heartwood of A. luederitzii, (**46**)- and (**48**)-Proguibourtinidin carboxylic acids (3,7,4-trihydroxy functionality) of 2,3-trans-3,4-trans-2,3-cis and 2,3-trans-3,4-trans-2,3-trans-configuration (such as 56), epicatechin or (+)-catechin as constituent units, and their related dimeric homologs, exist [102]. Certain condensed tannins from the *Acacia* subgenus Aculeiferum species were also investigated. Gallocatechin-(4 → 8)-epicatechin is present in *A. suma* bark [103]. Meanwhile, *A. galpinii’s* dark-brown heartwood is nearly tannin-free and contains teracacidins, a whole range of 7,8,4-trisubstituted flavonoids and melacacidin (7,8,3,4-tetrahydroxyflavan-3,4-diol), which is in common with many Australian species [93]. Several similar agents exist in the closely associated species of *A. burkei* [93]. The wood of *A. galpinii* contains -2,3-cis-3,4-trans-, -2,3-trans-3,4-cis-, and -2,3-trans-3,4-cis-Teracacidin, which are associated with melacacidin [-7,8,3,4-tetrahydroxy-2,3-cis-flavan-3,4-cis-dio. Remarkably, no tannins, except for proteracacinidin-type oligomers, exist in this species [93]. Their synthesis might result from the monomeric flavan-3,4-diol precursors’ pyrogallol A-ring losing its nucleophilicity, allowing alternate centers to take part in the formation of the interflavanyl bonding process [104]. These agents are complemented by flavonol, dihydroflavonol, flavanone, proteracacinidin dimers, and chalcone analogs [105]. Profisetinidins having a 2,3-cis comparative structure are uncommon and limited to certain proteracacididins from *A. caffra* and *A. galpinii* and a promelacacinidin from *A. melanoxylon* (subgenus Phyllodineae) [104]. *A. mearnsii*, (earlier known as *A. mollissima* or A. decurrens var. mollis), was previously the source of prosperous industries in Australia [106]. Nowadays, *A. mearnsii* represents the base of the industrial production of tannin p in Brazil and South Africa. Often 20–40% tannins occur in the bark of this specie, and the bark extracts contain approximately 70% PACs (up to 3000 MW) [107]. Because of its importance, extensive r investigations have been undertaken for the tannins of this species compared to others. The PACs contain essentially bi-, tri-, and tetrameric profisetinidins [fisetinidol- (4 → 8)- and -(4 → 6)-catechin profisetinidins], and an equivalent sequence of prorobinetinidins [107]. With 3,4,7-trihydroxyflavan-3-ol extender components, these profisetinidins are highly significant condensed tannins of trade, materializing the main elements of both quebracho (Schinopsis spp., Anacardiaceae) and wattle (*A. mearnsii*) tannins. The stereochemistry of naturally existing oligomers is usually 2,3-trans, with absolute configurations of 2S, 3R or 2R, 3S. Many other Australian wattle species have PACs with a (4β→8)-linkage and a (+)-catechin terminal unit. [93]. The PAC dimers are recurrent in Australian species with 7,3,4-trihydroxy subunits [(+)-mollisacacidin group]. Tannins are infrequently found in species of the 7,8,4-trihydroxy [teracacidin group], whereas tannins associated with the 7,8,3,4-tetrahydroxyflavonoids appear to form as oxidation products after isolation.

## 3. Pharmacological Activities

Many studies on the pharmacological properties of *Acacia* species have been conducted as a result of their widespread use in disease care. Secondary metabolites from the *Acacia* species have a variety of biological actions, including antibacterial, antifungal, antioxidant, anticancer, antiparasitic, antidiabetic, immunomodulatory, and cytotoxic properties (Table 1).

### 3.1. Anti-Hypertensive and Anti-Spasmodic Activity

A methanol (MeOH) extract from *A. nilotica* pods inhibited the rate and force of spontaneous shrinkages in the atria of paired guinea pigs. The activity of the pod extract is thought to be promoted by the calcium channel barrier since it suppressed K-induced contractions in rabbits. The extract’s potential was investigated using phentolamine and atropine. Although phentolamine inhibited nor-vasoconstrictor epinephrine’s action, the pretreatment of the extract with animal models had no influence on the NE effect, ruling out the role of adrenoceptor blocking. [9]. A methanol extract from the bark of *Acacia leucophloea* was investigated for potential spasmolytic effects on a rabbit jejunum preparation that spontaneously contracted due to its historical use as a gastrointestinal medication. The findings indicated that the spontaneous contractions were inhibited, pointing to a spasmolytic activity of *Acacia leucophloea* [108].

### 3.2. Anticancer and Anti-Mutagenic Properties

Plant-derived drugs have demonstrated the greatest impact in the antitumor field, with drugs such as vinblastine, vincristine, taxol, and camptothecin improving chemotherapy for some cancers [109]. A realistic and promising strategic approach to cancer prevention is the continuous search for new anticancer compounds in plant medicines and traditional foods [110]. Therefore, the quest for anticancer molecules in plant biodiversity continues, and *Acacia* species have not yet escaped this wave of research into natural anticancer molecules. *A. modesta* has a variety of pharmacological properties, including anticancer. In a study, the anticancer activity of the liver cancer cell line HepG2 was evaluated. In *A. modesta* plant extracts, HPLC analysis revealed the presence of phytochemicals, such as steroids, alkaloids, phenols, flavonoids, saponins, tannins, anthraquinone, and amino acids. The *A. nilotica* extract and its components exhibited antimutagenic/antiproliferative effects. The key agents are believed to be flowers and gums. In the investigation of [111], the anticancer potential of the gum (aqueous (Aq.) extract), leaves, and flowers were accessed on papillomagenesis, induced by 7,12-dimethylbenz(a) anthracene (DMBA). The total chromosomal was reduced in mice fed with an Aq. extract via oral gavage, in addition to the significant decrease in the occurrence of micronuclei to combat Dalton’s ascetic lymphoma (DAL); the mice models were pretreated with an A. nilotica extract with 10 mg/kg.b.w (14 successive days). The extract significantly reduced tumor expansion compared to the control group. The histopathological effects of the pre-treatment completed with the *A. nilotica* extract against acetaminophen-induced liver injury in rats showed that the injuries produced by acetaminophen were considerably reduced by owing to its ability to decrease the oxidative stress of acetaminophen-induced hepatocellular impairment [112]. The MeOH had a dose of 5 mg/plate, and the methanol extract from the bark reduced the UV-induced mutagenicity in E. coli WP-2. This decrease could be due to an enzymatic action that reversed pyrimidine dimmer synthesis. [113]. Furthermore, catechin-rich *A. catechu* extracts demonstrated anticancer activity against the human breast adenocarcinoma cell line (MCF-7) by inhibiting the expression of the transcription factors NF-B, p53, and AP-1, as well as nitric oxide levels [114]. The anticancer activities of A. catechu have already been well summarized previously [115]. Other species with anticancer properties are shown in Table 1.

### 3.3. Analgesic and Anti-Pyretic Activity

Several pathological diseases are triggered by inflammation and pain. The synthetic drugs used to treat these conditions have extremely toxic side effects. Global efforts are underway to introduce novel medicinal plants in order to develop effective, affordable, and safe drugs. Afsar and his colleagues investigated the anti-inflammatory, antipyretic, and analgesic activity of *A. hydaspica* methanol extract and its active fraction. The findings suggest that the existence of bioactive chemicals in *A. hydaspica* may be responsible for the pharmacological activities, validating the indigenous value of *A. hydaspica* towards inflammatory illnesses [12]. The analgesic effects of *A. nilotica* were evaluated in addition to the acetic-acid-mediated discomfort in rats (with 150 and 300 mg/kg b.w. as dose levels). The effective activity was displayed comparably to a standard drug, acetylsalicylate [116]. The hot-plate method was used to evaluate the analgesic effect compared to 1 mL of aspirin (100 mg/Kg) as a positive control. Organic acids, polysaccharides, and flavonoids were reported to be the inducers of the extract activity [117]. In an additional study, the analgesic and antipyretic effects were evaluated using brewer’s yeast (15%) with hot-plate methodology using Wistar Albino rats). The dose of the extract was 400 mg/kg b.w. and the results showed a reduction in the temperature of the rectum from 39.0070.25 °C to 37.7070.15 °C after administration (23 h) [118]. Furthermore, in another study, the analgesic, antipyretic activity, and anti-inflammatory potential of the methanolic extract obtained from *A. cyanophylla* were evaluated [119]. By using the hot-plate method, the analgesic activity showed a maximal activity of 36.98%, and similar patterns were seen for antipyretic activity.

### 3.4. Anti-Inflammatory

Many studies have proposed the anti-inflammatory activities of different species of *Acacia*. Eldeen and his coworkers [120] investigated the anti-inflammatory properties of acassane diterpene niloticane extracted from an ethyl acetate bark extract of *Acacia nilotica* subsp. Kraussiana. The results showed that in the cyclooxygenase test, niloticane possessed activity with IC50 values of 28 and 210 microM against COX-1 and COX-2, respectively. Moreover, the anti-inflammatory potential of using TPA-induced ear edema in mice was presented by the bioactive compounds obtained from aerial portions of *Acacia nilotica* [121]. The outcomes of the inhibitory activities of the pretreatment undertaken with *A. nilotica* extract (Aq.) on yeast-induced pyrexia and carrageenan-induced paw edema in rats (with a dosage of 100 mg/Kg b.w.) exhibited a reduction in the edema of the paw (20%), relatively less than that shown by 1 mL aq. aspirin solution (47%) [117]. Aq. pod extracts (50 and 100 mg/kg b.w.) were investigated in rats for carrageenan-induced edema, and the cotton pellet-induced granuloma models and the results showed a maximum inhibition of 64.41 percent and 25.62 percent, respectively [122]. Other groups of researchers investigated the anti-inflammatory properties of extracts from the heartwood of *A. confuse*. They found that *A. confusa* heartwood extracts or derived phytocompounds have a high potential for preventing diseases induced by the increased production of reactive oxygen species, including inflammatory diseases [13]. In an additional study, *A. tortilis*, an Algerian Sahara plant, was evaluated for its phenolic composition and biological activities. The biological activity of the *A. tortilis* extract was noteworthy, and the phenolic compounds discovered were proposed to be a useful starting point for the creation of cytotoxic and anti-inflammatory medicines [14]. Previous research has also shown that the pharmaceutical properties of *A. hydaspica* might be attributed to its indigenous value against inflammatory diseases [12]. The extracts of *Acacia modesta* also showed potent anti-inflammatory, antipyretic, analgesic, antidepressant, and anticoagulant activities, which conclude that the bark of *A. modesta* has significant therapeutic potential [123].

### 3.5. Anti-Microbial Activity

The species of *Acacia* have broad-spectrum antibacterial properties against a variety of diseases. *A. ataxacantha* is a pharmacological species widely used in the traditional medicine of the Benin Republic to treat infectious disorders. Three chemicals (**1**–**3**) were isolated and identified from the bark of *A. ataxacantha* using chromatographic and spectroscopic techniques. Three triterpenoids were isolated during a phytochemical analysis of *A. ataxacantha* (Fabaceae) (lupeol (**1**), betulinic acid (**2**), and betulinic acid-3-trans-caffeate (**3**). Compound 3 had a higher MIC value of 12.5 g/mL against *Staphylococcus epidermidis* and *Candida albicans*. The overall findings of this investigation showed that compound 3 isolated from *A. ataxacantha* has antimicrobial activity towards Gram-positive and Gram-negative bacteria and yeast, particularly *Candida albicans* [16]. *A. plicosepalus* yielded a novel flavanocoumarin, loranthin (**1**), as well as catechin (**2**), quercetin (**3**), rutin (**4**), gallic acid (**5**), and methyl gallate (**6**). Loranthin has the unusual flavanocoumarin skeleton, which connects the flavan and coumarin moieties via C-7/C-8 of both moieties. Loranthin’s antibacterial activity was tested against many pathogens, and it was found to have a substantial impact against Staphylococcus aureus [18]. The other *Acacia*, i.e., *A. farnesiana*, has been shown to exhibit antibacterial activity against *Vibrio cholerae*. By using nuclear magnetic resonance (NMR) methods ((**1**) H NMR and (**13**) C NMR), the active component was extracted from *A. farnesiana* and identified as methyl gallate. Methyl gallate degraded cell membrane integrity, resulting in a reduction in cytoplasmic pH (pHin, from 73 to 30) and membrane hyperpolarization, and the treated cells no longer generated ATP [19]. Cavazos and his coworkers evaluated the antibacterial and antioxidant activity of the extracts of *A. berlandieri* and *A rigidula* leaves (acetone, methanol, and acetic acid) [23]. *A. rigidula* leaf extracts exhibited antibacterial activity against *Pseudomonas alcalifaciens*, *Pseudomonas aeruginosa*, *Y. enterocolitica*, *E. coli*, *S. aureus*, and *E. faecalis*, while *A. berlandieri* displayed minimal inhibitory effects against *P. alcalifaciens*, *P. aeruginosa*, and *Y. enterocolitica*; thus, highlighting the significance of the species as a antimicrobial agent. The microbial inhibition of *A. nilotica* (leaf extract) on *E. coli* was proposed to be associated with the antibacterial activity, with a minimum inhibitory concentration of 70 mg/mL. This demonstrated the plant’s potential for treating Campylobacter-related microbial infections. [38]. The susceptibility of Campylobacter to the extract is of great interest seeing the common antibiotic resistance phenomena of the organism [38]. The aqueous extracts of *A. catechu* also exhibited an antimicrobial effect against *Staphylococcus aureus*, *Pseudomonas aeruginosa*, *Proteus mirabilis*, *E coli*, and *Klebsiella pneumonia* with a diameter of zone of inhibition (ZoI) of 17.66 ± 1.52, 16.66 ± 1.15, 14.0 ± 2.0, 8.33 ± 0.57, and 8.0 ± 0.0 mm, respectively [124]. Many other studies proposed the potent antimicrobial activity of *A. catechu* [125,126,127]. Thus, the data suggest that *A. catechu*, which is abundant in bioactive secondary metabolites, may be a promising source of compounds for the development of new antimicrobial drugs.

### 3.6. Antioxidant Activity

*Acacia* species contain a high concentration of polyphenolic chemicals, which have significant antioxidant capabilities and help to reduce the risk and treatment of oxidative stress-related disorders, such as cardiovascular, neurological, and cancer. Several publications have extensively studied the antioxidant activity of *Acacia* species in vitro, utilizing radicals such as 2,2-diphenyl-1-picrylhydrazyl (DPPH) and 2,2’-azino-bis[3-ethylbenzothiazoline-6-sulphonic acid] (ABTS) [128]. *A. hydaspica* is a natural shrub that has a variety of medicinal characteristics. Afsar et al. evaluated *A. hydaspica* polyphenol-rich ethyl acetate extract against cisplatin (CP)-induced lung-damaged rats. The results showed that because of its inherent antioxidant capacity and polyphenolic components, *A. hydaspica* extract might serve as a possible adjuvant that reduced CP-induced lung damage [17]. Lotfi Ghribia and his coworkers investigated the anti-acetycholinesterase and antioxidant potential activity of compounds and extracts from *A. cyanophylla*. In the DPPH test, the ethanolic extract of the flowers exhibited the highest antioxidant effect (67.26 µg/mL). The isolated compound, Isosalipurposide 1, also showed a significant antiradical power [21]. Next, five *Acacia* heartwood methanolic extracts, including *A. mangium, A. auriculiformis, A. crassicarpa, A. leucophloea*, and *A. deccurens*, were tested for antioxidant activity using three different in vitro assays. The *A. crassicarpa* extracts exhibited the highest antioxidant activity, which was accompanied by high phenolic and hydrolyzable tannin concentrations [22].

Many countries around the world, including Pakistan, consider soil salinity to be a severe environmental issue. *A. stenophylla* and *A. albida* were studied in a hydroponic experiment to investigate distinct mechanisms of salinity resistance. According to the findings of this study, *A. stenophylla* is more salinity-tolerant than *A. albida* because it maintains better ionic balance and stronger antioxidant enzyme activities, resulting in increased biomass production [129]. Additionally, the flower extracts of A. dealbata were investigated during three stages of flowering. The results indicated that the hydroethanolic extracts performed well for all of these biological activities, including antioxidant activity, and the results varied according to the maturity status of the flowers, with the early stage being the most active, which can be attributed to the chalcones content [25]. These first results suggested that *A. dealbata* could be a good source of natural antioxidants, which may be used to stabilize free radicals that induce oxidative stress.

In another study, during the MeOH extract fractionation of the *A. nilotica* extract, the AN-2 fraction was isolated and recognized using spectroscopic methods, i.e., mass spectroscopy and NMR, to be a derivative of coumarin (umbelliferone). The anti-oxidative properties in vitro, comprising the deoxyribose, DPPH, reducing power, lipid peroxidation, and chelating power assays, were performed. The results showed that the anti-oxidative activity of umbelliferone was dependent on the concentration, almost 100 μg/mL, and leveled off later with no more activity enhancement. This shows the first document for the antioxidant activity exhibited by umbelliferone isolated from *A. nilotica* [38]. In the study, the two extraction techniques were evaluated for radical scavenging power. The results showed that the consecutive extraction was efficient in concentrating the bioactive agents in the extract when compared with the maceration. The investigation indicated that the extract of ethanol has high quantities of flavonoid and phenolic compounds and had effective and significant antioxidant potential. The antioxidant activity of the extract (ethanolic) may be attributed to its ability to donate electrons or hydrogen, as well as its direct radical scavenging abilities [130]. Similarly, different studies reported the antioxidant potential of *A. catechu*. Aryal et al. found that both ethanol and methanol extracts of *A. catechu* barks exhibit antioxidant potential, assessed by DPPH radical scavenging assay [131]. Additionally, DPPH radical and ABTS radical scavenging assays, ferric reducing power assays, super-oxide radical scavenging assays, and lipid peroxidation with an IC50 of 48.65–54.44 mg of equivalents/g powder demonstrated the antioxidant capacity of methanol and aqueous extracts of *A. catechu* [132].

### 3.7. Anti-Filarial and Antidiabetic Activity

Diabetes is one of the world’s five main causes of death. Many herbal medications have been advocated for the treatment of diabetes in addition to the currently available therapeutic options [128]. Previously, authors have documented Acaciaside (both A and B), glucuronic, and methylglucuronic acid, rhamnose, galactose, and arabinose isolated from many parts of *A. auriculiformis* (Fabaceae) [133]. Acaciaside A and B, which was isolated from the funicles, displayed anti-filarial property both in vitro and in vivo vis à vis bovine filarial parasite *Setaria cervi* [134,135]. The *A. auriculiformis* ethanolic extract was found to be effective against microfilaria and tested on dogs infected with *Dirofilaria immitis* [28]. Acaciaside A also has anti-cestocidal activity [136]. It is also documented to possess anti-plasmodial activity [137]. The *A. nilotica* ssp. Indica was found to display hypoglycemic properties and the blood sugar levels dropped by almost 25.05% in normal rats nourished for one week; however, it did not exhibit significant hypoglycemic activity in alloxanised diabetic rats. The hypoglycaemic property of the legumes was owing to the indirect or direct stimulation of the β–cells of islets of Langerhans to release more insulin [138]. The other study suggests that a hydroethanolic leaf extract of *A. auriculiformis* possesses good antioxidant and enzyme inhibitory potential, which, in turn, might be responsible for its antidiabetic effects [29]. Similarly, *A. catechu* extracts showed an antidiabetic activity towards porcine pancreatic *α*-amylase, [139] with an IC_50_ of 49.9 μg/mL. Ethyl acetate, dichloromethane, aqueous fractions, and a crude methanolic extract of the bark of *A. catechu* showed the inhibitory activity of α-glucosidase and α-amylase, with IC50 ranges of 9–115 g/mL, in another investigation [131]. Kumar et al. studied the antidiabetic potential of an aqueous extract of *A. tortilis* polysaccharide from gum exudates and its role in the comorbidities associated with diabetes in STZ-nicotinamide-induced diabetic rats [140]. The administration of 500 and 1000 mg/kg doses of the extract showed a significant reduction in fasting blood glucose level compared to diabetic control after 7 days, thus, revealing the potential of *A. tortilis* for the treatment of T2DM and its comorbidities. Furthermore, chloroform extracts of *A. arabica* bark significantly decreased the elevated serum glucose levels in alloxan-induced diabetic albino rats [141].

### 3.8. Antiviral and Nematicidal Activity

The crude extract of the plant’s leaves presented antiviral effects against the *Turnip mosaic* virus in vitro. The reduction was found in a number of lesions on the host’s *C. album* (80.2%) and *Chenopodium amaranticolor* (93.77%), and the lesions decreased once the extract was present on the leaves of the host. The potato virus was repressed by the bark extract [142]. The nematicidal activity was observed by the Aq. leaf extract of the plant and *A. senegal* as well against *Meloidogyne incogenita* by the inhibition of its hatching [143]. DNA viruses, viruses belonging to Herpesviridae, Poxviridae, and Papillomaviridae were targeted using *V. nilotica*, and resulted in anti-bovine herpes virus and direct virucidal activity against goatpox virus. The meta-analysis inferred that *V. nilotica* could be a promising source of anti-hepatitis C virus drug leads with the ability to prevent its long-term sequelae while promoting immune competence [144]. Additionally, another study aimed to assess the cytotoxic and antiviral activity of an aqueous leaf extract of *A. catechu* against the new castle disease virus (NDV) on human peripheral blood mononuclear cells (PBMC). Variable doses of the aqueous leaf extract of *A. catechu* (0.5–30 mg/mL, 50 L; diluted in phosphate-buffered saline, PBS) were tested, and the results showed that at higher concentrations, the aqueous leaf extract of *A. catechu* suppressed NDV proliferation and also decreased CD14 monocyte surface marker with or without NDV [31].

## 4. Pharmaceutical Preparation That Have *Acacia* its Main Molecules

The prospective, placebo, randomized, and positively controlled test was intended to assess the clinical properties of an existing gel enclosing *A. arabica* for decreasing gingival inflammation and plaque in gingivitis patients. Ninety individuals diagnosed with chronic general gingivitis were included, and then they were arbitrarily distributed (into three groups): Group I, constituted the placebo; Group II, gumtone; Group III, chlorhexidine (1%). A clinical assessment was carried out using the plaque index at baseline and the gingival index of Loe and Silness. Gumtone gel presented substantial clinical enhancement against the index of plaque and gingival scores in comparison to Group I, with a placebo-containing gel. The enhancement was similar to 1% chlorhexidine (gel). Dissimilar to chlorhexidine gel, the gumtone gel was not related to unpleasant taste or the discoloration of teeth [145,146].

### 4.1. Auromere’s Ayurvedic Formula, Name of Brand: Auromere Ayurvedic, Item: 327045-172138

This combines the ordinary teeth whitening fiber, PEELU, which exhibits invigorating and astringent characteristics of NEEM, as well as other roots, barks, flowers, and plants that were valued by Ayurvedic Professionals for centuries for their distinct and combined efficacy in maintaining superior hygiene. Polishes and cleans teeth to their whitest, refreshes and soothes sensitive gums, and purifies the breath and mouth naturally. Ingredients: Pomegranate Rind Glycerin (from vegetable oil), Fine Chalk (a gentle cleanser), Water, Bishop’s Weed (flower extract), Carageenan (seaweed), Silica, Peelu (*salvadora persica*), Indian Almond, Sodium Lauryl Sulphate (coconut oil (india)), Sarsaparilla, Rose Apple, silica, Indian Licorice Root, Neem (*azadirachta*), Clove, Common Jujube, Bark of Barleria Prinoitis (vajradanti), Persian Walnut, Asian Oak, Bedda Nut, Sappan Wood, *Zanthoxylum alatum* (tejbal), Catechu, Prickly Ash, *A. arabica* Bark (babul), Bengal Madder, Cinnamon, Mayweed, Medlar Bark, carageenan (from seaweed), cellulose gum (from plants), sodium lauryl sulphate (from the Indian coconut oil), Spearmint Oil, Cinnamon Bark Oil, Eucalyptus Oil, Peppermint Oil, Menthol, Anethole, and Thymol.

### 4.2. Glyconutrient Powder, Name of Brand: Now Foods, Item: 355483-186295

Immunity maintenance supports intercellular interactions, which are offered through the glyconutrient blend and eight sugar immune enhancers. Glyconutrient Blend is a branded blend of ingredients. It contains eight immune-boosting glyconutrients derived from the whole coffee bean, as well as other natural ingredients. These nutrients are known as vital components for cellular network signaling, as well as playing particular functions in immune responses. ImmunEnhancer™ (a polysaccharide) is derived from a Larch known as Arabinogalactan. The basis of good health is built upon a strong responsive immune system. Arabinogalactan’s effects on NK Cells have been shown i to maintain a healthy immune system in scientific investigations.

### 4.3. Powder of Organic Acacia Fiber, Name of Brand: Now Foods, Item: 415 723-206319

Organic *Acacia* Fiber Powder is a dietary fiber made from the gum of the *Acacia* tree; it is pure, natural, and soluble. Research has indicated that soluble fiber in the diet can aid in improving bowel regularity. The powder also exhibits tremendous prebiotic potential, as it is able to support healthy intestinal flora. Since it reduces fermentation and decreases bloating and gas problems, *Acacia* powder is well tolerated. This powder can be taken on a daily basis and does not contain any gastrointestinal irritant stimulants [147]. To date, scientists are looking for new alternatives and ways to increase the value of the various products derived from *A. nilotica*. The by-products of the species *A. nilotica* have been explored for their full use in diverse fields. The multipurpose value of *A. nilotica* as a source of fodder, fence posts, fuel, wood, shade, gums, and tannin has provoked the researchers with further directions for investigation. The gum obtained from the trunk has already been part of the cosmetic, textile dyeing, printing, food, and pharmaceutical industries [148]. The leaves have also been used to remove hazardous synthetic colors and heavy metals from wastewater as bio-adsorbents. It can also be used as a bio-indicator to monitor pollution from heavy metals, copper, and cobalt.

## 5. Herb–Drug Interaction

To date, research based on drug–herbal interactions has generally been limited to case reports and limited systematic reviews. Meanwhile, for the treatment of several medical conditions, supplements of herbal drugs are used around the world. These are used in combination with drugs or used alone [149]. In some cases, the combined use of herbs and drugs may alter the pharmacokinetic profile of drugs [150]. The simultaneous usage of herbal medicines may increase, oppose, or stimulate the activity of drugs. Clinically, the lack of efficacy might be the consequence of these interactions, as well as toxic reactions, and unexpected side effects. Thus, healthcare professionals should remain vigilant for the potential interactions between herbal medicines and prescribed drugs, especially when drugs with a narrow therapeutic index are used. The data show that the *Acacia* gum obtained from *A. rabi*, reduced the absorption of drugs, such as amoxicillin [151]. The findings indicate that the coexistence of amoxicillin and gum rabic in the upper gastrointestinal tract resulted in a pharmacokinetic interaction that significantly decreased the absorption of amoxicillin. The other study proposed that black catechu can significantly alter theophylline pharmacokinetics in vivo, possibly due to the inhibition of CYP1A and P-glycoprotein activity; thus, precaution should be exercised when administering black catechu with CYP1A substrate [152].

## 6. Conclusions

Ethnopharmacology is the field that involves the detailed study of plants to identify new medications and the development of advanced drugs. Genus *Acacia* includes a group of plants with promising pharmacological properties. This review summarizes the presence of phytoconstituents in different *Acacia* species with diverse and potent pharmacological properties, including anti-inflammatory, analgesic, antioxidant, antimutagenic, antiproteolytic, antiviral, nematicidal activity, and so on. Mainly, polyphenols are responsible for the antioxidant potential of different extracts and the said pharmacological potential. The use of *Acacia* extracts and their active agents in different herbal formulations further demonstrated the medicinal value of this genus. Due to the promising therapeutic role of different species of *Acacia*, further studies should be carried out in order to develop multifunctional drugs and describe their bioavailability, pharmacokinetics, physiological pathways, and importance to human health in sufficient detail.

## Figures and Tables

**Table 1:** Biochemical components and pharmacological properties of different species of genus *Acacia*.

*Acacia* Species	Plant Part Used	Extraction Solvents	Phytochemicals	Pharmaceutical Action	Reference
*Acacia nilotica*	Pods/Leaves	Methanol, Ethanol	Phentolamine	Antihypertensive, Antispasmodic, Antimicrobial	[9,10]
*Acacia modesta*	Whole plant	Ethanol	Steroids, Tannins, Phenols, Alkaloids, Saponins, Flavonoids, Anthraquinone	Anticancer	[11]
*Acacia hydaspica*	Bark/twigs/leaves	Methanol	Polyphenolic	Antipyretic, Anti-inflammatory, Analgesic	[12]
*Acacia confusa*	Heartwood	Ethanol	Flavonoids (i.e., 7,3′,4′-trihydroxy-3-methoxyflavone) Melanoxetin (3,7,8,3′,4′-pentahydroxyflavone)	Anti-inflammatory	[13]
*Acacia tortilis*	Leaves	Ethanol	flavan-3-ols galloyl	Cytotoxic, Anti-inflammatory	[14]
*Acacia arabica*	Leaves	Ethanol	Flavonoids, Terpenoids, Tannins	Abortifacient	[15]
*Acacia mellifera*	Stem bark	Methanol	3-(Z)-trans coumaroylbetulin, 3-(E)-cis coumaroylbetulin	Antimalarial	[15]
*Acacia ataxacantha*	Stem bark	Hexane, Dichloromethane, Ethyl acetate Methanol	betulinic acid, betulinic acid-3-trans-caffeate	Antimicrobial, Antioxidant	[16]
*Acacia* *hydaspica*	Aerial parts	Methanol	polyphenol	Antioxidant	[17]
*Acacia plicosepalus*	Whole plant	Methanol	loranthin, quercetin	Antimicrobial	[18]
*Acacia farnesiana*	Barks	Hexane, Chlorofom, Methanol	methyl gallate	Antimicrobial	[19]
*Acacia arabica*	Leaves/bark	Methanol	quercetine 3-O- (4′-O-acetyl)-rhamnopyranoside	Antioxidant	[20]
*Acacia cyanophylla*	Flowers	Ethyl acetate	naringenin	Antioxidant	[21]
*Acacia crassicarpa*	Heartwood	Methanol	quercetin, 5,7,2′,5′-Tetrahydroxyflavone	Antioxidant	[22]
*Acacia rigidula*	Leaves	Acetone, Methanol, Acetic acid	diterpenes, tannins	Antimicrobial, Antioxidant	[23]
*Acacia* *albida*	Leaves/bark	Ethanol	polyphenol	Antioxidant	[24]
*Acacia dealbata*	Flowers	Ethanol	acetylcholinesterase	Antioxidant	[25]
*Acacia saligna*	Leaves	--	myricetin-3-O-α-l-rhamnoside, quercetin-3-O-α-l-rhamnoside	Antioxidant, Cytotoxic	[26]
*Acacia karro*	Leaves	Ethyl acetate, Chloroform	β-sitosterol, epigallocatechin	Antilisterial	[27]
*Acacia* *auriculiformis*	Whole plant/Leaves	Ethanol, Hydroethanolic Extract	saponins, 2,4-dinitrophenyl salicylic acid	Antifilarial, Antidiabetic	[28,29]
*Acacia pennatula*	Pods	Methanol	4-nitro-O-phenylenediamine	Antioxidant, Antimutagenic	[30]
*Acacia* *catechu*	Leaves	Aqueous,		Cytotoxic, Antiviral	[31]
*Acacia aroma*	Leaves	Ethanol	5-diphenyl tetrazolium bromide	Antibacterial, Cytotoxic	[32]

**Table 2:** Analytical data of the gum exudates from *Acacia* trees.

*Acacia* Species	Moisture Content (%)	Ash Content (%)	Nitrogen Content (%)	Total Protein (%)	pH	Molecular Weight ×10^6^	Viscosity cm^3^ g^−1^	Sp. Rot	Acid Equivalent Weight	Glucouronic Acid%	References
*Acacia nilotica* var. *tomentosa*	5.80	0.04	0.10	0.62	4.48	-	-	+80.16	-	-	[65]
*Acacia nilotica*	-	-	-	4.7	-	-	35	+21	-	21	[66]
*A. nilotica*	-	2.48	0.02	-	-	2.2	9.5	+108°	1890	9	[67]
*Acacia senegal* var. *senegal*	-	3.32	0.37	2.4	-	-	15.4	−31.5	1153.8	16.8	[68]
*Acacia seyal* var. *seyal*	-	2.43	0.14	0.95	-	-	11.6	+61	1185.8	16.4	[68]
*Acacia senegal* var. *senegal*	-	4.89	0.35	2.3	4.78	-	18.9	−30	1620	11.89	[69]
*Acacia seyal* var. *seyal*	-	4.47	0.22	1.4	5.16	-	15.5	+52	1180	16.34	[69]
*Acacia nilotica* var. *nilotica*	10.81	1.91	0.02	0.16	5.15	-	10.19	+99.17	1908.37	10.18	[70]
*Acacia senegal* var. *senegal*	13.49	3.27	0.35	2.31	-	-	14.61	−32	1161	15.2	[71]
*Acacia senegal* var. *senegal*	9.76	3.40	0.33	2.16	4.94	0.24	-	−31.75	-	-	[68]
*Acacia seyal* var. *seyal*	9.56	2.50	0.63	4.16	4.53	2.01	-	+48.25	-	-	[68]
*Acacia mellifera*	8.35	3.13	0.24	1.61	4.84	2.95	-	+56.00	-	-	[68]
*Acacia tortilis* var. *raddiana*	8.49	2.05	1.55	10.34	4.45	2.06	-	+86.75	-	-	[72]
*Acacia catechu*	13.9	03.4	-	04.8	4.59	-		−28.17			[73]

## Data Availability

Data presented in this study are available on reasonable request.
